# Holographic Writing of Ink-Based Phase Conjugate Nanostructures *via* Laser Ablation

**DOI:** 10.1038/s41598-017-10790-4

**Published:** 2017-09-06

**Authors:** Muhammad Waqas Khalid, Rajib Ahmed, Ali K. Yetisen, Bader AlQattan, Haider Butt

**Affiliations:** 1 Nanotechnology Laboratory, School of Engineering, University of Birmingham, Birmingham, B15 2TT UK; 2 Harvard-MIT Division of Health Sciences and Technology, Harvard University and Massachusetts Institute of Technology, Cambridge, MA 02139 USA

## Abstract

The optical phase conjugation (OPC) through photonic nanostructures in coherent optics involves the utilization of a nonlinear optical mechanism through real-time processing of electromagnetic fields. Their applications include spectroscopy, optical tomography, wavefront sensing, and imaging. The development of functional and personalized holographic devices in the visible and near-infrared spectrum can be improved by introducing cost-effective, rapid, and high-throughput fabrication techniques and low-cost recording media. Here, we develop flat and thin phase-conjugate nanostructures on low-cost ink coated glass substrates through a facile and flexible single pulsed nanosecond laser based reflection holography and a cornercube retroreflector (CCR). Fabricated one/two-dimensional (1D/2D) nanostructures exhibited far-field phase-conjugated patterns through wavefront reconstruction by means of diffraction. The optical phase conjugation property had correlation with the laser light (energy) and structural parameters (width, height and exposure angle) variation. The phase conjugated diffraction property from the recorded nanostructures was verified through spectral measurements, far-field diffraction experiments, and thermal imaging. Furthermore, a comparison between the conventional and phase-conjugated nanostructures showed two-fold increase in diffracted light intensity under monochromatic light illumination. It is anticipated that low-cost ink based holographic phase-conjugate nanostructures may have applications in flexible and printable displays, polarization-selective flat waveplates, and adaptive diffraction optics.

## Introduction

The optical phase conjugation (OPC) is a complex phenomenon of a photorefractive medium in which a phase conjugated wave (PCW) is generated^1^. The PCW is the reverse phase of an electromagnetic field at every point as compared to incident light^2^. It has applications in optical tomography, interferometry, near-field microscopy, wavefront correction, imaging, and biochemical sensing^2–5^. The PCW can be generated *via* three major mechanisms. The first method involves four wave mixing process; the second technique is based on various backward simulated (Brillouin, Raman, Rayleigh-wing or Kerr) scattering processes; and the third method is pivoting on single/multi-photon pumped backward stimulated emission (lasing) process. There is a common physical mechanism among all three techniques which plays the same vital role in generating backward phase conjugate beam which is based on optical nonlinearity^6–10^. Optical nonlinear interaction is generally responsible for OPC^2^. An ideal phase conjugate mirror (PCM) reflects a light wave in which the wave vector and amplitude are reversed and are complex conjugated to the incident wave to create PCW^6,7^. A CCR also produces phase conjugated reflected light through total internal reflection (TIR) based on three-mirror reflection effect^11^. However, the use of three dimensional or volumetric PCMs or CCRs are limited due to fabrication complexity, time-consuming processes, and high cost. Recently, flat optical devices have been produced on functionalized substrates using low-cost single pulse holography^12,13^. Additionally, optical phase-conjugated flat CCR array holograms have been created in functionalized gelatin substrates through holographic Denisyuk reflection mode recording^11,14^.

Holography is an optical process to record and reconstruct amplitude and phase information by means of diffraction through monochromatic or broadband light illumination. It has applications in data storage, image display, security, and biochemical sensing^15–23^. The word “hologram” can be referred to the physical structure as well as to the resultant diffraction pattern^24–26^. An off-axis hologram is recorded by projecting an interference pattern consisting of scattered light from an object and a coherent reference beam^27,28^. Fabrication methods in holography include surface stamping/imprinting (*i.e*. embossing), focused ion beam (FIB) milling, and electron beam lithography (EBL) to produce nanoscale resolution patterns^29–32^. However, these methods are complex, expertise dependent, high-cost, and time-consuming^12^. To address these challenges, direct laser writing has been developed to produce 2D/3D complex structures and off-axis holograms using mask-free laser lithography^33,34^. Recently, direct laser writing based on single pulse Denisyuk reflection holography have been utilized to produce optical devices through selective removal of light absorbing materials through nanosecond laser ablation^12,13,27,35^. Single pulse Denisyuk reflection holography is a low cost, fast, and simple technique to fabricate nanophotonic devices^12,36^.

Here, we develop a flexible, low-cost and simple Denisyuk reflection image writing method to record phase conjugate diffraction holograms. A single pulsed nanosecond laser (1064, 90–300 mJ) was used to record phase conjugate holograms on ink coated glass substrates. In this process, an incident laser beam from Nd:YAG laser propagates through the light absorbing material coated on a glass substrate and is reflected back from the object (CCR) placed perpendicular to the incident beam propagation direction. Conjugated periodicity depends on the intensity distribution of an interference pattern and optical properties of light absorbing material (*i.e*. absorption, refractive index). Resulting conjugated patterns are also affected by the tilt angle of the recording media (θ), working distance (d), height (h), and localized energy (E) distribution from the laser source. Therefore, 1D/2D phase conjugate nanostructures have been produced in ink-substrates through optimized parameters. Fast-Fourier transform (FFT) simulation of the recorded samples were performed to predict the conjugated diffraction patterns. Optical characterization of nanostructures were performed through monochromatic (red, green, and violet) and broadband light at normal illumination. The projection experiments through an image screen setup allowed displaying phase-conjugated diffraction patterns at the far-field. The diffracted wavefronts showed symmetrical and phase-inversion from non-diffracted specular reflection. As compared to conventional gratings, conjugated surface patterns showed two-fold increase in diffraction intensity at monochromatic red light normal illumination.

## Results and Discussion

### Phase Conjugate Nanostructure Recording

**Figure 1:**
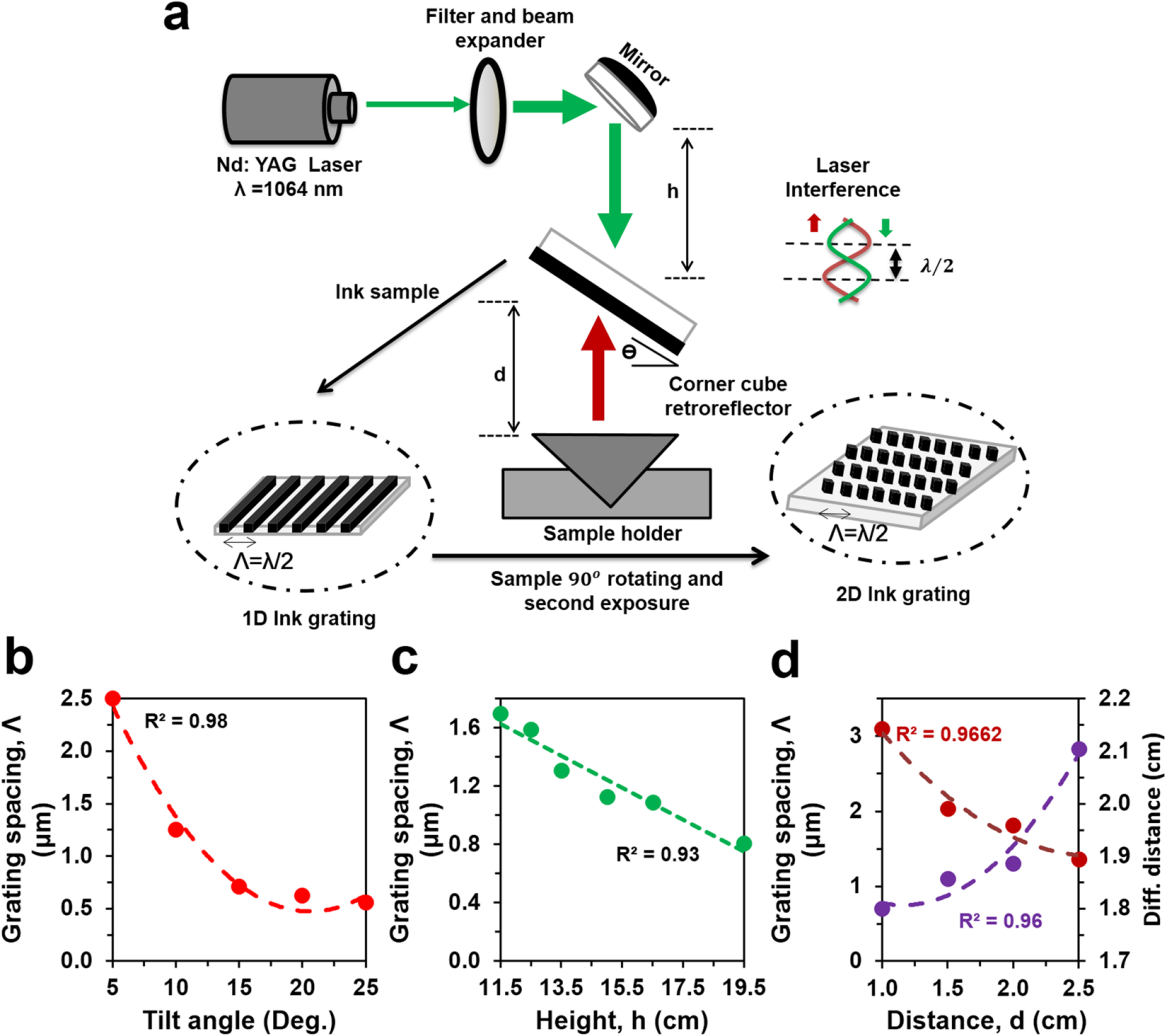
Holographic fabrication of phase conjugate nanostructures using Denisyuk reflection mode recording on ink coated glass substrate. (**a**) Schematic of the experimental setup to fabricate conjugate nanopatterns. (**b**) Grating spacing as a function of tilt angle (*θ*°), *i.e*. the angle between the ink coated glass substrate and the surface plane of CCR. (**c**) Grating spacing as a function of height (*h*), *i.e*. the distance between laser tip and the surface plane of CCR. (**d**) Grating spacing of the structures and separation between the diffraction spots of resulted diffraction pattern as a function of working distance (*d*), *i.e*. distance between ink-coated glass substrate and CCR.

The optical recording of the phase conjugated nanostructures was based on a single pulsed nanosecond laser and in-line Denisyuk reflection holography (Fig. 1a). A 1064 nm beam from a Nd:YAG laser (pulse duration = 3.5 ns, 300 mJ) passed through optical filters, a beam expander and series of dichroic mirrors setup before exposing the recording medium. At the exposure stage, a recording medium (black ink coated on a glass slide) and an object (single corner cube retroreflector) were placed parallel to each other. The expanded laser beam passes perpendicular to the exposure stage and reflected back from the object. The laser beam entered in the CCR from the surface plane, reflected once from each three surfaces of the CCR, finally, phase conjugated with respect to the incident beam. Both incident and reflected beams propagated in opposite direction. The resulting standing wave propagated toward the substrate, ablated the ink thin film to produce periodic gratings. The standing wave was produced as a result of interference between the incident and reflected waves allowed forming ablated and non-ablated regions on the recording medium as shown in Fig. 1a.

Sample preparation was based on the following stages: (a) glass plate cleaned with acetone, dried for 1 min, (b) black permanent ink was diluted in ethanol (1:1, v/v), (c) ink-ethanol solution was poured on to the glass plate, spinning at 400 rpm for 60 s, followed by 900 rpm for 20 s, and (d) ink-coated glass sample was exposed to the laser pulse for recording nanostructures (as shown in Supporting Information Figure S1a). The recording process was completed in few seconds. The transmission spectrum of the coated black ink indicated broadband absorption in the visible range (Supporting Information Figure S1b,c).

For materials exposed to nanosecond laser pulses, the principal mechanisms of material removal are vaporization and phase explosion^37–39^, which are discussed in detail by Lutey *et al.*^40^.

### Conjugate Nanostructure Recording Theory

Phase conjugate nanostructures were copied in the form of periodic gratings on an ink coated glass substrate *via* laser ablation. The recording process involved light inference through a corner cube retroreflector in a light sensitive ink-based holographic recording medium. A corner cube retroreflector having three mutually perpendicular mirror surfaces that reflect light toward the source through total internal reflection. The incident light is reflected three times to create a retroreflection^5^. However, not all light entering to a cornercube reflected three times and become a part of retroreflection. Light entering at the center of the cornercube has more probability to be retroreflected than entering at the sides (Supporting Information Figure S2a,b)^11^. The retroreflected light is phase conjugated to the incident light. The incident light, transmitted (plane-to-plane of CCR) and the reflected phase conjugated light interferes and produces phase conjugated nanostructures at the ink substrate through laser ablation. For an arbitrary incident plain wave under paraxial approximation is:

1 $${E}_{i}(r,t)=\varepsilon A(i){{\rm{e}}}^{[i.({k}_{i}.r-\omega t)]}$$

where $A(i)$ is a amplitude function, ${k}_{i}$ is a wave vector, and $\varepsilon$ is a unit polarization vector so that $\varepsilon \,.{\varepsilon }^{\ast }=1$. The light reflected from a CCR can be approximated with ideal PCM if (a) the physical dimension of the CCR is large enough, and (b) the center of the CCR coincide with optical axis. Therefore, reflected wave from a CCR is:

2 $${E}_{CCR}(r,t)=R{\varepsilon }^{\ast }{A}^{\ast }(i){{\rm{e}}}^{[i.(-{k}_{i}.r-\omega t)]}$$

where R indicates amplitude reflectivity of a CCR, −${k}_{i}$ shows reverse direction of the reflected wave, ${\varepsilon }^{\ast }$, and ${A}^{\ast }(i)$ indicates conjugate/reversal of polarization and amplitude vectors^41^. The recorded phase-conjugated nanostructure due the resulting complex interference between the incident and transmitted or reflected PCW is:

3 $$E(r,t)={E}_{i}(r,t)+{E}_{CCR}(r,t)=[\varepsilon A(i){{\rm{e}}}^{i.({k}_{i}.r)}+R{\varepsilon }^{\ast }{A}^{\ast }(i){{\rm{e}}}^{i.(-{k}_{i}.r)}]{e}^{-i\omega t}$$

Considering total electric field, ${E}_{i}(r,t)$ in half-space, $z > 0$, time-average field can be approximated as $\langle E\rangle =(1/16\pi )E\,.{E}^{\ast }$^42^. Therefore, from Eq. (3) it can be expressed that:

4 $$\langle E(r)\rangle =(1/16\pi )\{(1+{|R|}^{2}){|A(i)|}^{2}+[{\varepsilon }^{2}{R}^{\ast }A{(i)}^{2}{e}^{(2i{k}_{i}.r)}+CC]\}$$

where CC denotes complex conjugate. Using trigonometry identity and $R=|R|{e}^{i\varphi }$, $A(i)=|A(i)|{e}^{i\alpha }$, ${\varepsilon }^{2}=|{\varepsilon }^{2}|{e}^{i\delta }$, the interference field is ref.^41^:

5 $$I=\langle E(r)\rangle =\frac{{|A(i)|}^{2}}{16\pi }[1+{|k|}^{2}+2|k||{\varepsilon }^{2}|\times \,\cos (2{k}_{i}.r+2\alpha -\phi +\gamma )]$$

Therefore, for maximum light interference, $\cos (2{k}_{i}.r+2\alpha -\phi +\gamma )=0$ and

6 $${I}_{\max .}=\frac{{|A(i)|}^{2}}{16\pi }[1+{|k|}^{2}+2|k||{\varepsilon }^{2}|]$$

For minimum light interference, $\cos (2{k}_{i}.r+2\alpha -\phi +\gamma )=-\,1$ and

7 $${I}_{\min .}=\frac{{|A(i)|}^{2}}{16\pi }[1+{|k|}^{2}-2|k||{\varepsilon }^{2}|]$$

For simplicity, the interference between the incident and retoreflected light was considered. Interference due to transmitted light from plane-to-plane of CCR was not considered. However, phase-conjugated nanostructures through a CCR is a complex multibeam interference phenomenon between the incident, reflected (without three time reflection) from different planes, and retoreflected beams.

### Ink-Based Conjugate Nanostructures

Phase conjugate 1/2D nanostructure were fabricated on an ink coated glass substrate through in-line Denisyuk reflection holography. Figure 1a shows the experimental diagram to produce ink-based conjugate nanostructures. 1/2D phase conjugate nanostructure were based on single or two pulses of laser beam(s) at specific exposure angles. The fabricated nanostructures were uniform to produce conjugate patterns (Supporting Information Figure S2c). The characteristics of ink-based nanostructure patterns depend on laser wavelength, energy, and geometrical parameters. Ink-coated glass substrate was adjusted in a flexible sample holder (able to move along x, y and z axes) above the retroreflector to vary the tilt angle (*θ*) of the substrate from 0° to 25°, distance, *d* from 1.0 cm to 2.5 cm with respect to the surface plane of the retroreflector and also height, *h* the distance between Nd:YAG laser tip to the surface plane from 11.5 cm to 19.5 cm. Moreover, Nd:YAG laser energy, *E* was varied from 90 to 360 mJ. During nanostructure recording, only one parameter was changed at one time and other parameters were kept constant. Figure 1b–d depicts the spacing of fine diffraction conjugate grating structures as function of *θ*, *h*, and *d*.

The grating spacing decreased from 2.50 μm to 0.555 μm with increasing tilt angle of the glass substrate from the surface plane (*θ* = 5°,10°, 15°, 20°, 25°) while keeping other parameters constant (*E* = 300 mJ, *h* = 11.5 cm and *d* = 1.5 cm). Similarly, as the *h* and *d* increased, the grating spacing decreased (Fig. 1c,d). For observing the influence of *h* variation on grating spacing, all other parameters were kept constant, *E* = 300 mJ, *d* = 1.5 cm, and *θ* = 15°; whereas for *d* variation, *h* was fixed at 11.5 cm, *E* = 300 mJ, and *θ* = 15°. Additionally, the resolution of diffraction i.e. distance between the diffraction orders increased with decreasing *d*.

### Optical Characteristics of 1D Conjugate Structures

**Figure 2:**
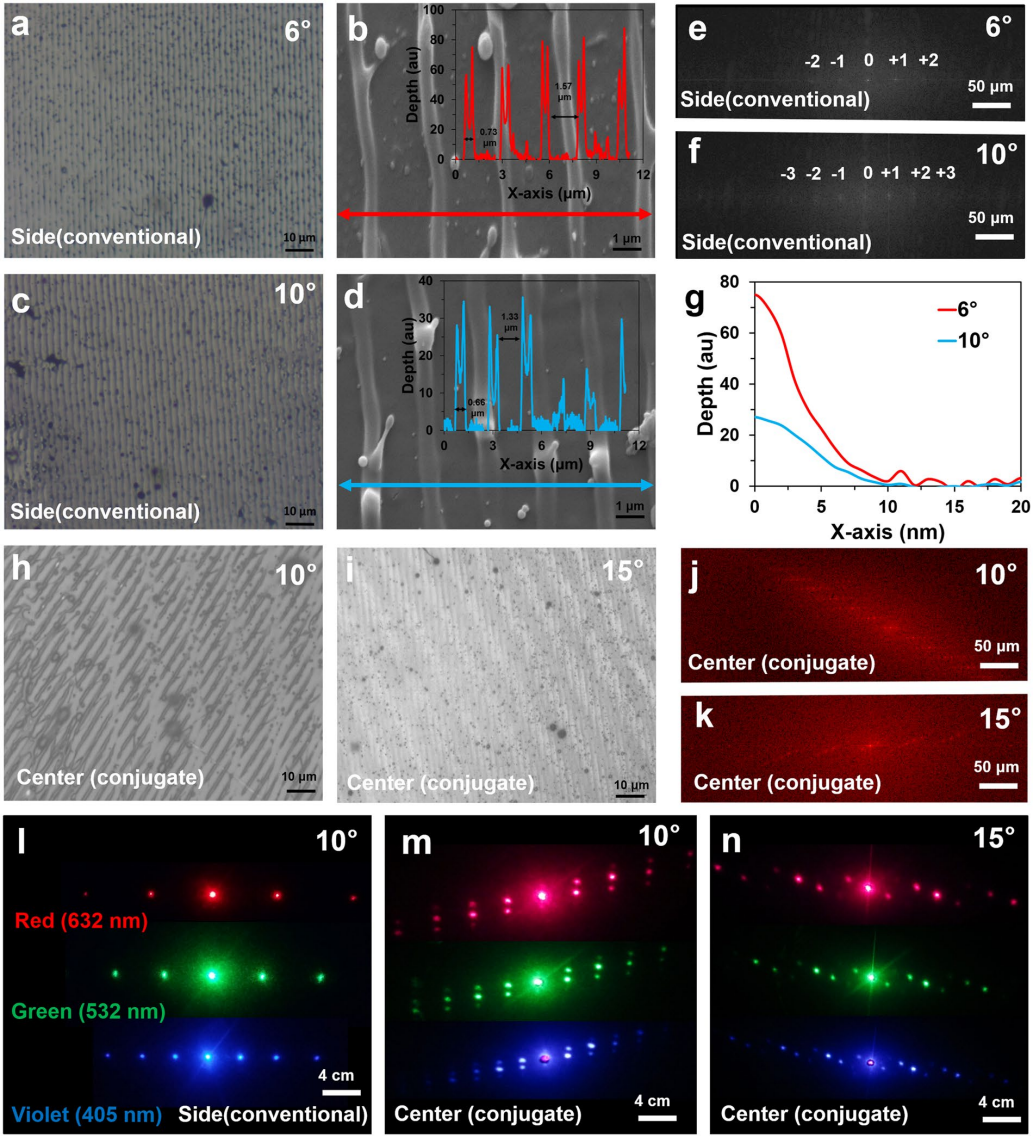
Surface morphology and response toward monochromatic light illumination of the conventional and conjugated nanopatterns. (**a**–**d**) Optical and electron microscope images of the conventional gratings recorded at 6° and 10° tilt angles. (**e**,**f**) FFT of the planar patterns with 6° and 10° tilt angles. Scale bars = 50 μm. (**g**) Depth of the normal fabricated patterns at 6° and 10° tilted sample. (**h,i**) 2D phase conjugate nanostructures recorded by tilting sample at 10° and 15°. (**j**,**k**) FFT of the conjugated patterns with 10° and 15° sample tilt angles. Scale bars = 50 μm. (**l–n**) Projected conventional, and conjugated diffraction patterns through red (λ = 650 nm), green (λ = 532 nm), violet (λ = 450 nm) light in normal transmission. Scale bars = 4.0 cm.

The recorded patterns in the ink-based 1D conjugate nanostructure were analyzed through microscopy. The light entering near the sides of the CCR does not show three mirror retroreflection effect and acts as a normal mirror. Only light beam entering at the active regions of CCR undergoes three mirror reflection and produce retroreflection properties. Therefore, the recorded nanostructures with sidewall reflection were analogous to plane mirror reflection based holographic recording. The resulting recorded nanostructure (diffraction grating) was based on two-beam interference strategy. Figure 2a–d shows optical and scanning electron microscope images of the recorded diffraction gratings at the sidewall laser illumination regions of the CCR with different tilt angle variations (θ = 6° and 10°). The computed Fast Fourier Transform (FFT) of the recorded nanostructures allowed visualizing 1D diffraction patterns (Fig. 2e,f). As the tilt angle increased, the diffraction distance also increased due to the decrease in grating spacing. Lowering the tilt angle increased the ablation depth (Fig. 2g). However, light entering at the active regions of the CCR showed retroreflection. Light enters from one plane of CCR to another with 45° before leaving the last plane and returning toward the source. Therefore, light entering at the active regions of the CCR produces multibeam interference to create conjugate diffraction gratings. Figure 2h,i show optical microscopic images of the conjugate nanostructures. The multigratings were formed due to multiple inference beams between the incident and phase conjugate waves from different CCR planes. The large grating structure was due to the light interference between the incident light and reflected phase conjugate light and having 180° interference angle. However, internal tilted smaller grating structure was due to the light interference between incidents light and reflected light during passing from one plane to the others and having an interference angle of 45° (Fig. 2h). The electron microscope image of the conjugated nanostructure also had larger and internal smaller multigrating structures (Supporting Information Figure S2d–f).

**Figure 3:**
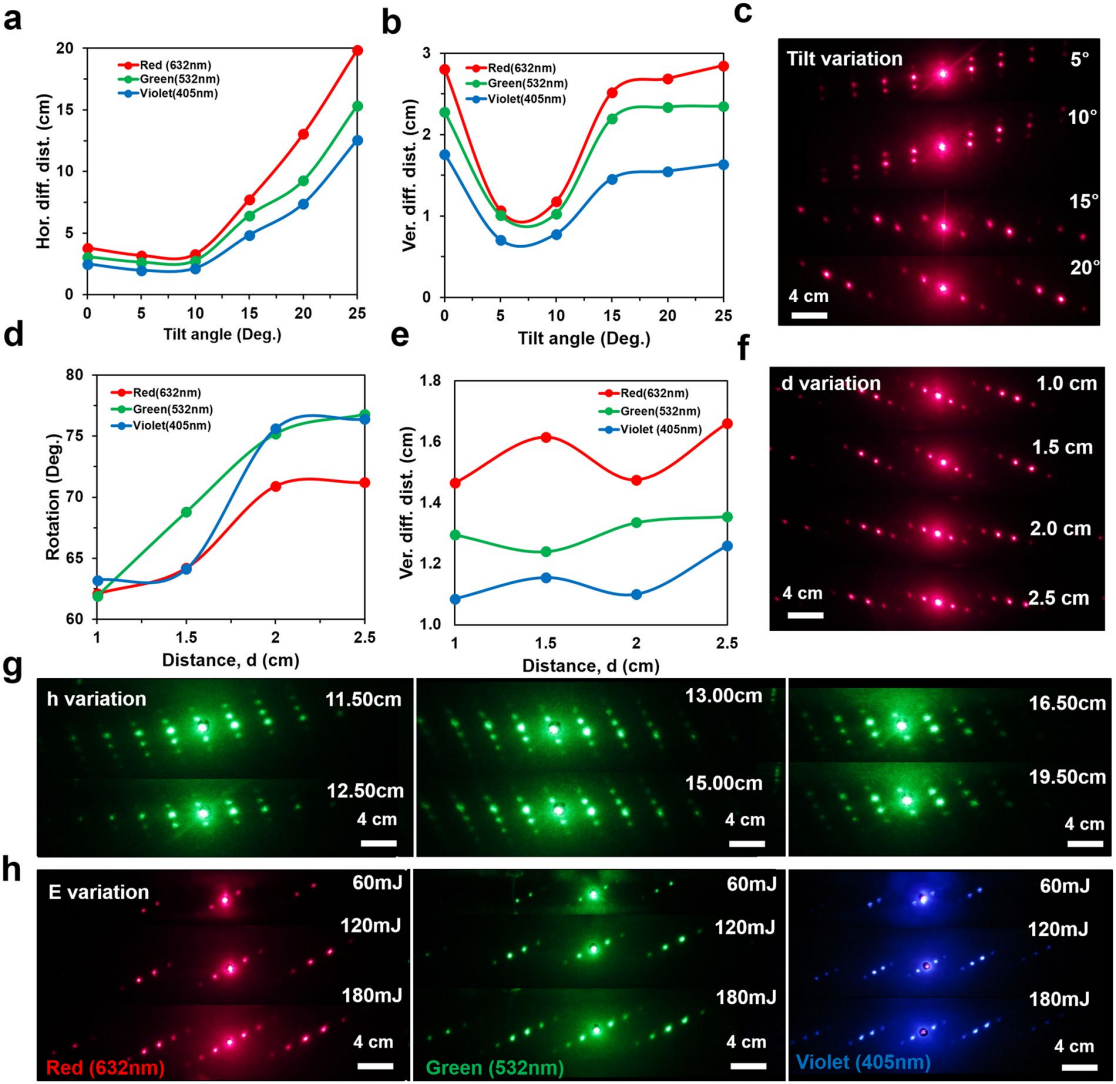
Optical characterization of 1D phase conjugate diffraction patterns with respect to variations in optimized parameters. (**a,b**) Horizontal and vertical diffraction distance as a function of tilt angle (*θ*) variation. (**c**) Far-field diffraction patterns through tilt angle variation at normal red light illumination. Scale bar = 4.0 cm. (**d,e**) The rotation angle and horizontal diffraction distance as a function of distance (*d*) variation. (**f**) Far-field diffraction patterns through distance (*d*) variation at red light normal illumination. Scale bar = 4.0 cm. (**g**,**h**) Far-field diffraction patterns through height (*h*), energy (*E*) variation using red, green, and violet light normal illumination. Scale bar = 4.0 cm.

The FFT simulation of the recorded conjugated nanostructures were predicted from the far-field diffraction patterns (Fig. 2j,k). The simulated FFT patterns showed tilted multi-order diffraction patterns when the illumination angle was varied. Furthermore, the far-field diffraction patterns of the fabricated planar and conjugated nanostructure were captured through the image screen experiment (Fig. 2l–n). The monochromatic (red, green, and blue) light was normally illuminated at the recorded nanostructures. Well-ordered symmetric diffraction patterns were observed. The diffraction patterns of the conjugated structures were different as compared with the simple structures. The 1^st^ order diffraction distances along vertical and horizontal directions were measured from the non-diffracted zero-order. As the tilt angle increased, the horizontal diffraction distance increased exponentially with red, green, and violet light at normal illumination (Fig. 3a,b). Similarly, generally vertical diffraction distances were also increased with variation in tilt angle. However, maximum diffraction distances were observed with red light illumination at tilted angles. Violet light showed minimum diffraction distance as compared with green light at tilted illumination angles. Therefore, the diffraction property of the conjugate nanostructure followed Bragg’s law (Λ = λ/2sinθ, where Λ is grating spacing, λ is incident wavelength, and θ is tilt angle). The far-field diffraction pattern with tilt angle variation (5–20°) was captured with normal red light illumination. Green and violet diffraction patterns were also captured as the tilt angle was varied (Supporting Information Figures S3, 4). Multiorder distinct diffraction patterns were observed due to conjugated nanostructures with tilted angle variations.

Diffraction patterns were also analyzed through working distance, *d* variation (Fig. 3c,d). The rotation angles of the vertically oriented orders from the normal (y-axis) were plotted in degrees as a function of working distance, *d* variation from 1.0 cm to 2.5 cm with 0.5 cm increment. As *d* increased, the rotation angles increased sinusoidally. The horizontal diffraction distances were also plotted as a function of working distance (*d*) variation. Red and blue light also showed maximum and minimum horizontal diffraction distances and increased sinusoidally as the working distance (*d*) increased. The far-field diffraction patterns were also captured with red light normal illumination at distance, *d* variation. Further conjugate diffraction patterns as a function of height (*h*) and energy (*E*) variation were also captured through monochromatic (red, green, and violet) light illumination (Fig. 3g,h).

### 1D Phase Conjugated Diffraction

**Figure 4:**
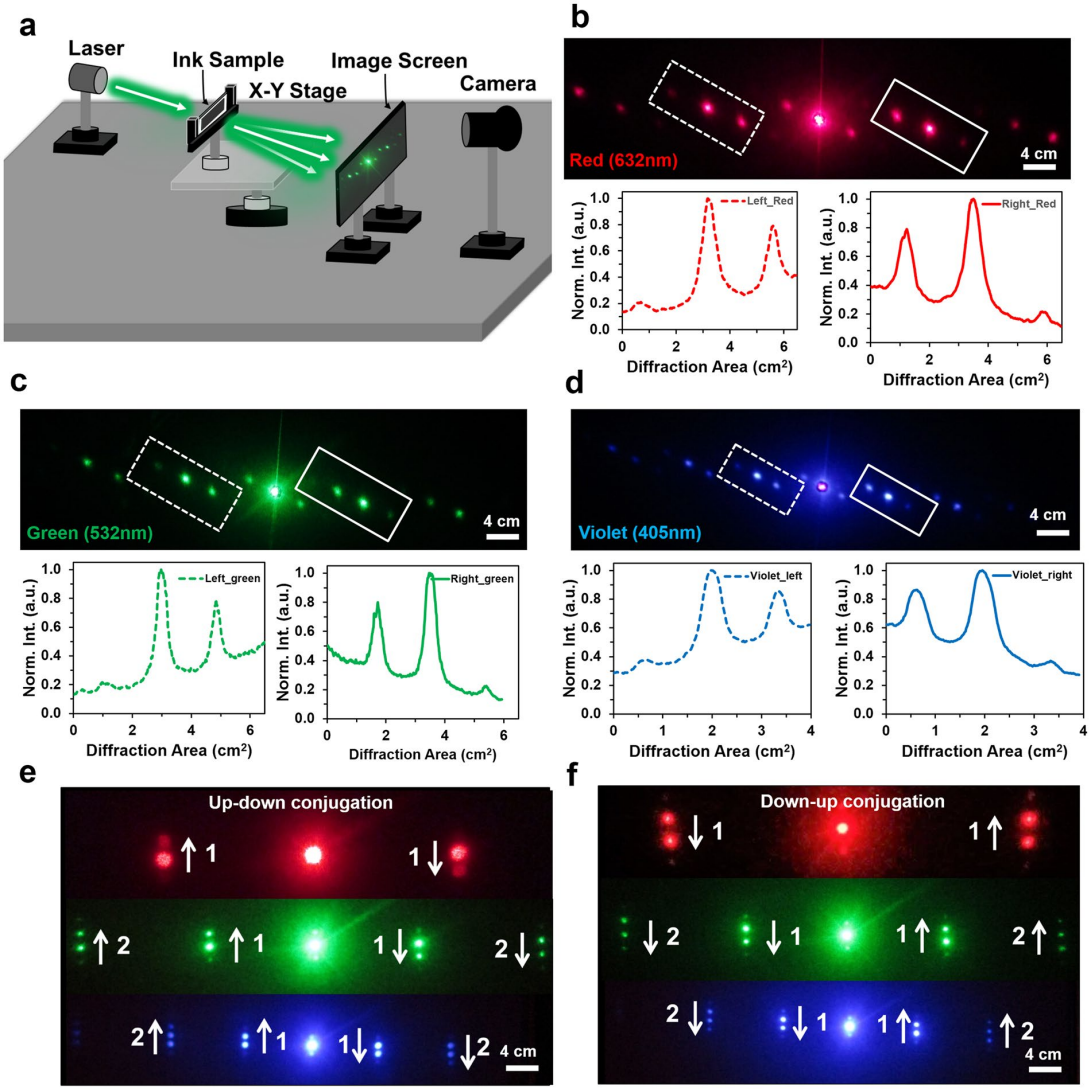
1D phase conjugate diffraction patterns through monochromatic (red, green, and violet) light normal illumination. (**a**) Schematic of the far-field image diffraction setup. (**b–d**) Far-field conjugate diffraction patterns (15° tilted structure) and relevant plot profiles showing phase conjugation (inversion symmetry) between first orders of red, green and violet diffraction patterns. Scale bars = 4.0 cm. (**e,f**) Far-field conjugate diffraction patterns (10° tilted structure) in vertical direction with monochromatic light normal illumination. Arrows indicate conjugation between the diffracted orders and associated numbers (1 or 2). Scale bars = 4.0 cm.

Far-field optical experiments were performed with 1D conjugated nanostructures through a screen setup for image projection (Fig. 4a). The monochromatic light was illuminated normally to a conjugate nanostructure fixed in a sample holder, which was positioned through a 2D x-y rotational stage. A white screen was placed behind the sample holder and a digital camera was used to capture the far-field patterns. Figure 4b–d shows the captured images of the conjugate diffraction patterns with red (λ = 650 nm), green (λ = 532 nm) and violet (λ = 440 nm) lights at 15° tilt angle. It is obvious from all three different colored conjugate patterns that the same order numbers along each side of the central spot are analogous in shape, but in reverse intensity order *i.e*. they have inversion symmetry. If a vector (R) is associated with the first order on the left hand side of the zero-order specular reflection spot, then the first order on the right hand side will be a vector (−R) *i.e*. same magnitude but in antiparallel orientation. This conjugate diffraction property is also applicable to the second order and other higher orders. Phase conjugation between same order number is obvious by measuring the patterns obtained with respect to the intensity of the spots. The intensity plots profiles in the insets were produced on the left (dotted line) and right sides (solid line) of the first order diffraction patterns. Both plots had similar trends, but oriented along the opposite direction, which indicates their mutual phase conjugation or inversion symmetry. The phase-conjugation patterns were analyzed through conjugated structure recorded at 20° tilt angle (Fig. 4e,f). Phase conjugation patterns in vertical direction were showed with arrows and order numbers. The conjugated diffraction order is always opposite to each other or inversely symmetric. 1D phase conjugate diffraction properties were analyzed with 15° and 20° tilted conjugate nanostructures. However, phase conjugated diffraction properties were also valid with other tilt angles (0–25°), laser energy (*E*) and structural parameter variation (*h* and *d*) (Supporting Information Figures S3–7).

### 2D Conjugated Nanostructure

**Figure 5:**
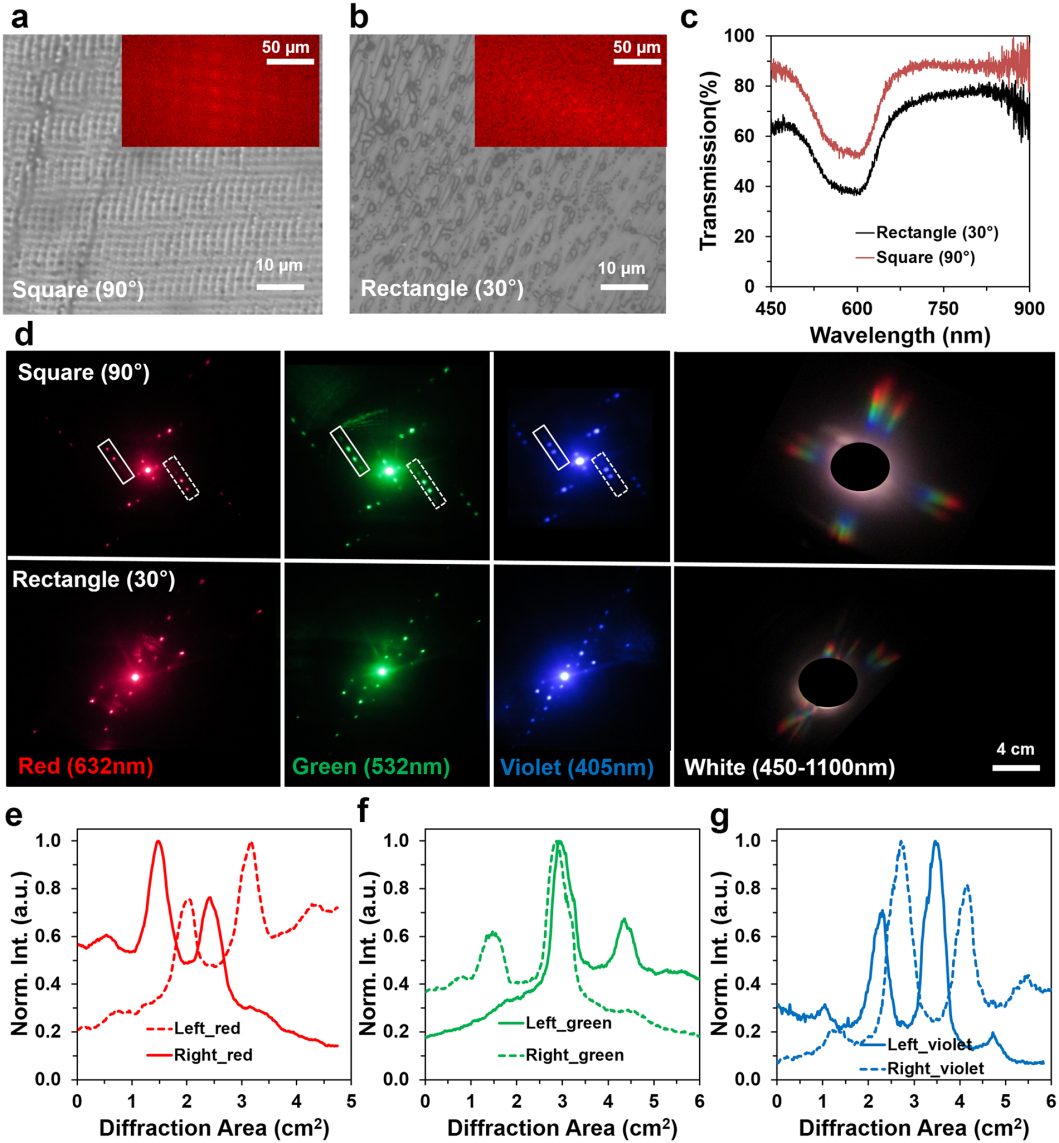
Ink-based 2D conjugated nanostructure fabrication using Denisyuk reflection holography. (**a,b**) Optical microscopic images of 2D conjugated nanostructures. Insets show FFT patterns. Scale bars = 10 μm. Inset scale bars = 50 μm. (**c**) Optical transmission property of the 2D square (90°), and rectangular (30°) conjugated structures. (**d**) Far-field diffraction patterns with monochromatic (red, green, and violet) and broadband light normal illumination. Scale bar = 4.0 cm. (**e–g**) Left and right side conjugated wavefronts (1^st^ order) through monochromatic light normal illumination.

2D conjugated nanostructures were fabricated by a two pulse laser exposure to the ink-based recording medium using the setup shown in Fig. 1a. After the first exposure, the sample was rotated to a predefined angle and subsequently exposed to a second laser pulse. Multiple pulses of different laser energies, and precise exposure angles are required to create customized complex nanophotonic structures. Square and rectangular conjugated nanostructures were fabricated with 90° and 30° rotation of the samples before second exposure. Figure 5a,b shows the optical microscopic images of the recorded ink-based 2D conjugated nanostructures. Square (90°) and rectangular (30°) internal microstructures were observed from the microscopic images. The simulated FFT of the recorded conjugate structures allowed predicting the far-field 2D square and rectangular patterns (insets in Figure S5a,b)^43^.

Optical characteristics of the 2D phase conjugated structures were studied with light transmission measurements through an optical microscope. The light transmission through the 2D rectangular (30°) structure was higher than a 2D triangular (90°) structure (Fig. 5c). However, the transmission property of the 2D structure was similar with black ink and 1D grating (Supporting Information Figure S1b,c). The monochromatic light (red, green, and violet) and broadband light normally illuminated the conjugate structures to produce far-field diffraction patterns (Fig. 5d). 2D diffraction patterns were observed with predefined recording angles. Diffraction spots obtained from the 90° square shaped grating were well defined and also positioned at 90° with respect to each other resulting into a square shaped 2D diffraction pattern for each color. In rectangular 2D conjugate diffraction pattern obtained from 30° rectangular gratings, diffraction spots were arranged in a rectangular pattern, but were not distinguishable by orders.

For broadband light illumination, square (90°) and rectangular (30°) far-field rainbow patterns were observed. Violet light diffracted at the lowest angle, green at intermediate and red at the highest angle as predicted from Bragg’s law (Fig. 5d). 2D phase conjugated diffraction property was also observed. Figure 5e–g shows profiles for 2D phase conjugation between the first orders on the left and right side of the central spot of squared diffraction patterns for red, green, and violet colors, respectively. Plots showed symmetrical phase inversion property at the left and right diffraction orders.

### Discussion

Phase conjugate diffraction gratings were fabricated through a cornercube retroreflector and nanosecond laser ablation in Denisyuk reflection holography mode. Recorded ink-based holograms diffracted light with phase conjugation similar to the function of a CCR. In the case of CCR, 3D geometry was involved during phase-conjugation, whereas the recorded gratings in ink had 2D geometry and showed phase conjugation properties in terms of 2D diffraction patterns under collimated light illumination. Conjugate nanopatterns were engineered by superimposition of standing waves in an ink medium coated on a glass substrate by changing recording parameters including working distance (*d*), height (*h*), tilt of the recording media (*θ*), and energy (*E*) of incident beam from the Nd:YAG laser. Fabricated nanostructures independently from the recording parameter variation diffracted light with phase conjugation under coherent/broadband light in transmission mode. The resultant conjugate diffraction patterns and diffraction behaviour were predicted by simulations.

**Figure 6:**
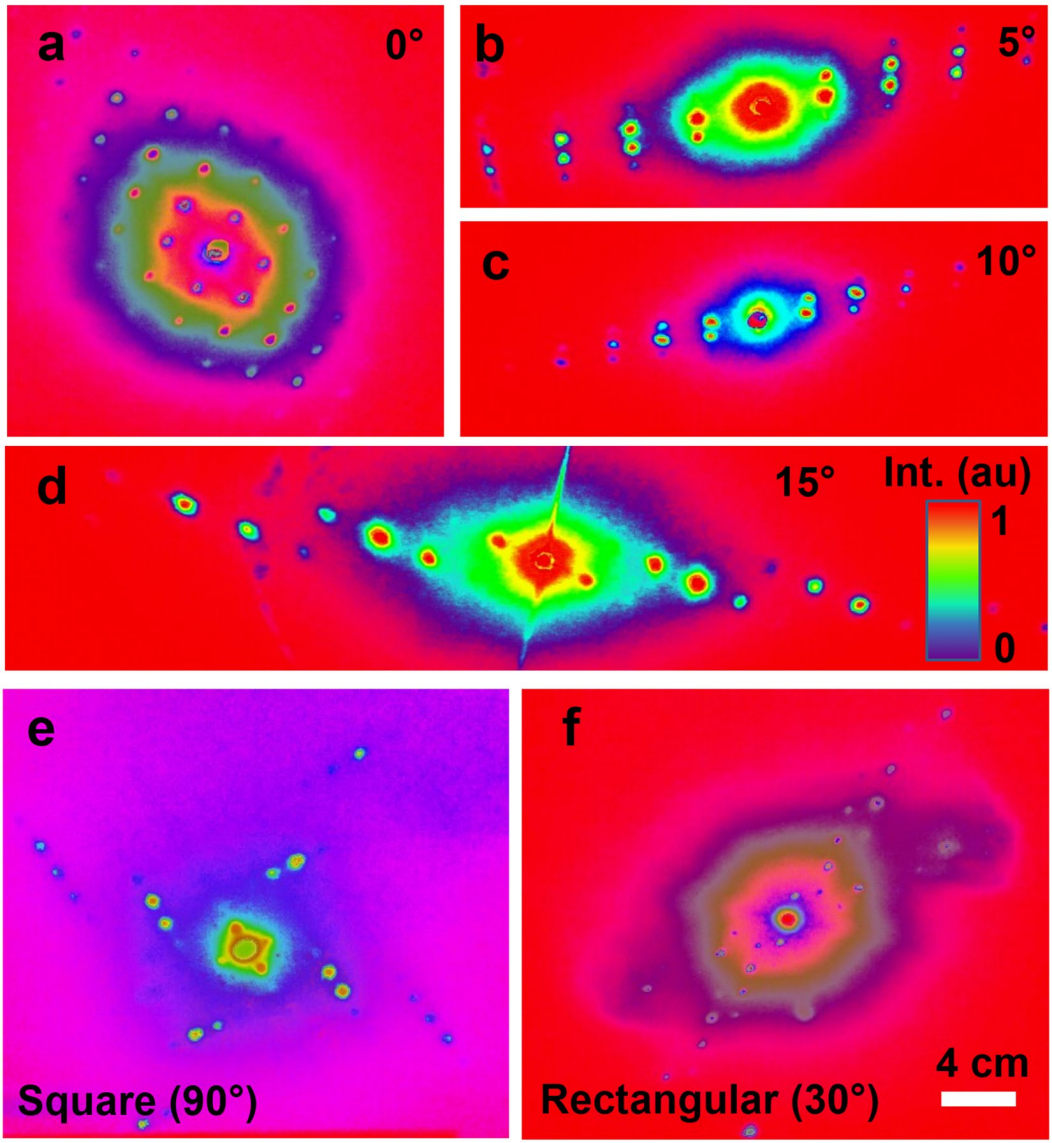
Thermal images of the ink-based 1/2D conjugated diffraction patterns. (**a**–**d**) Thermal 1D diffraction patterns with tilt angle (*θ*) variation. (**e**,**f**) Thermal images of 2D square (90°) and rectangle (30°) diffraction patterns. Scale bar = 4.0 cm.

Squared and rectangular 2D phase conjugate nanostructures were also fabricated by standing waves intersecting each other at superimposed exposure angles (90° and 30°). Multiple interference patterns were produced to fabricate 2D phase conjugate nanostructures by directing the incident beam toward the center of corner cube, where the incident beam splitted into smaller fragments. Each fragment reflected back toward the source, along the opposite direction with respect to the incident beam, resulting in multiple interference patterns. Only retroreflected standing waves contributed to the fabrication of the conjugate nanostructures, which had high-energy intensity to ablate the ink recording medium. Exposure parameters were varied to examine their influence on the characteristics of the recorded holograms in the form of periodic gratings and resulting images of the diffraction patterns in the far field. Optical phase conjugation was observed for both 1D and 2D ink-based nanostructures. The diffracted light intensity from the conjugated nanostructures were symmetrical. The phase-conjugated diffraction plots showed inverse symmetry between same order numbers on the left and right side with respect to non-diffracted zero-order. Thermal images of the 1D/2D diffraction patterns also showed symmetrical inversion properties of the light intensities (Fig. 6).

**Figure 7:**
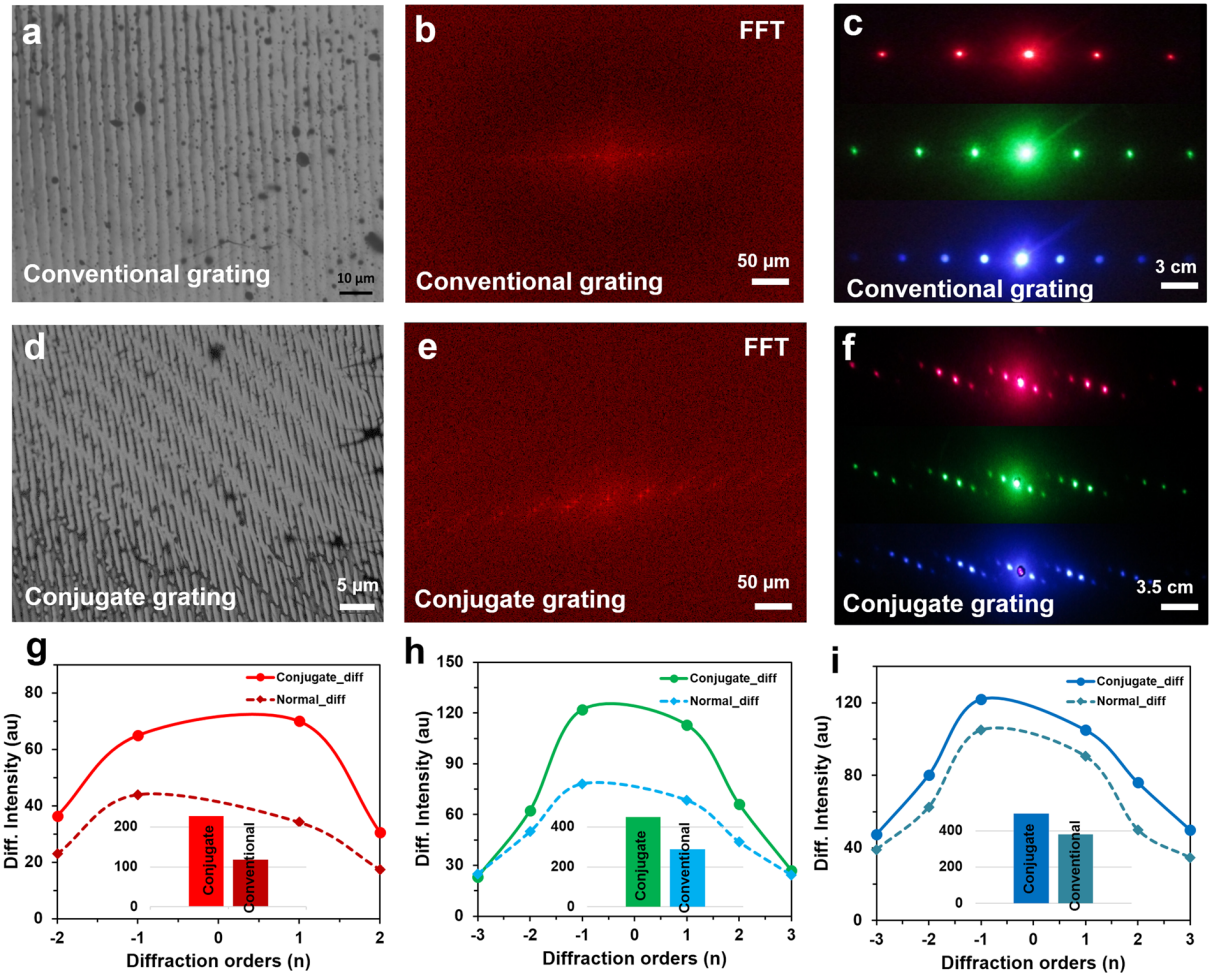
A comparison between ink-based conventional and phase conjugate nanostructures in terms of light diffraction. (**a**–**c**) Optical microscopic image of conventional ink-based nanostructure, FFT simulation, and light diffraction with monochromatic beam at normal illumination. Scale bars = 10 μm, 50 μm, and 3.0 cm, respectively. (**d**–**f**) Optical microscopic image of conjugated ink-based nanostructure, FFT simulation, and light diffraction with monochromatic light at normal illumination. Scale bars = 5 μm, 50 μm, and 3.5 cm, respectively. (**g–i**) Optical light diffraction of the conventional and conjugated nanostructures through red, green, and violet light at normal illumination.

Optical performance of the gratings were compared for the conventional diffraction spots and the phase conjugate diffracted spots. Figure 7a–f shows microscopic images of the ink-based conventional and conjugated nanostructures, its simulated FFT patterns, and far-field diffraction patterns through monochromatic light (red, green, and violet) at normal illumination. The diffracted light plots along the horizontal direction had similar optical properties (Supporting Information Figure S8). However, diffracted light intensities were compared between the conventional and conjugate orders (Fig. 7g–i). Phase conjugate nanostructures had higher efficiency of light diffraction than conventional gratings; red as 2:1, green as 2:1.2, and violet as 2:1.5. These nanostructures were fabricated by infrared laser beam (*λ* = 1064 nm) so the gratings diffracted red light more efficiently than green and blue lights. Measured horizontal distance between the first order and the central diffraction spot was for violet (min.) ~8.9 mm, green ~13 mm, and red (max.) ~18 mm during light transmission through conjugated nanostructures. Similarly, vertical mutual distance between the spots of first order was violet ~1.7 mm (min.), green ~2.1 mm, and red ~2.5 mm (max.) light transmission through conjugate gratings, respectively, which showed longer wavelength diffraction at higher angles. Therefore, maximum light diffraction from the ink-based conjugated nanopatterns may have possible applications in printable diffraction gratings and micro optical devices.

## Conclusion

We have developed a simple, and fast technique to obtain phase conjugated nanostructures by utilizing a single-step ND:YAG laser ablation method in holographic Denisyuk reflection mode. Fabrication of nanostructures using a corner cube retroreflector was efficient and robust, showing selective laser ablation though laser interference lithography on ink-coated glass substrates. Optical properties of the recorded 1/2D ink-based nanostructures showed multiple highly-intense diffraction orders, and more efficient optical phase conjugation. Light diffraction properties of the different orders from the non-diffracted zero-order were symmetric and showed efficient inversion of the wavefronts through monochromatic and broadband light at normal illumination. Moreover, the diffracted light intensity from the conjugated ink-based nanostructures showed almost two-fold efficient (at red light normal illumination) as compared to the conventional nanopatterns. Therefore, ink-based holographic nanofabrication has applications in printable low-cost optical devices include diffraction gratings, tunable wavelength-selective filters, diffusers, and lenses. Moreover, a single-step laser pulse ablation is a facile, reliable and efficient technique in terms of cost and device production time to record holographic patterns on various light absorbing materials including transparent/opaque polymers and dyes. Ink-based conjugate diffraction patterns having unique morphology with multiple features, which is a step forward in holography and they can be utilized to create numerous novel applications in encrypted data storage, identification (business cards), biosensing, and security labels.

## Materials and Methods

### Materials and Equipment

Black permanent ink (Staedtler, Germany) diluted with ethanol solution on a glass substrate was used as a reflection holography recording medium. The Nd:YAG laser operated at 1064 nm and 90–360 mJ of energy was used during holographic recording. Spectrophotometer (resolution 0.1 nm) and a broadband light source (450–1100 nm) were purchased from Ocean Optics for optical measurements. MATLAB (Math Works, v8.1) was used for the FFT simulations and data processing.

### Recording Medium Preparation

The sample production involved (i) acetone solution to clean the glass substrate, (ii) glass plate was left 5 min to dry and coated with black ink. Black permanent ink was diluted with ethanol (1:1, v/v) and was spin coated on the glass substrate (CHEMAT Technology, KW 4 A). Spin coating was performed at a speed of 400 rpm for 60 s, followed by 900 rpm for 20 s. The ink-coated glass plate was left to dry for 5 min, and was immediately ready for holographic recording.

### Ink-based Conjugate Hologram Recording

A CCR (N-BK7) was purchased from Edmund Optics, and used as an object during recording. Holographic recording of the conjugated nanostructures was based on multiple laser light interference between the incident, reflected (CCR face-to-face), and retroreflected beams. The ink-coated glass plate was used as a recording medium. Selective ablated and non-ablated regions were created in ink medium through laser ablation. Light incident to the side walls did not show retroreflection property and reflected light (compared to plane mirror) and produced conventional diffraction patterns. However, light incident to the middle region of the CCR (active regions) reflected three times, and conjugated. Phase conjugated structures were produced by the retroreflected light.

### Optical Characterization of the Gratings

A spectrophotometer, monochromatic and broadband light sources were used to optically characterise recorded nanostructures. The broadband light source was used during light diffraction measurements and produced rainbow colors through normal illumination at the hologram surface. The monochromatic light sources: red (635 nm, 4.5 mW, Ø11 mm), green (532 nm, 4.5 mW, Ø11 mm), and violet (405 nm, 2.6 mW, Ø11 mm) were purchased from Thorlabs Elliptec GmbH (Dortmund, Germany). Far-field diffraction experiments were performed through monochromatic and broadband light illumination through an image screen setup (white A4 paper).

## Electronic supplementary material

Supporting Information
