# Author Correction: Holographic Writing of Ink-Based Phase Conjugate Nanostructures *via* Laser Ablation

**DOI:** 10.1038/s41598-018-24169-6

**Published:** 2018-04-18

**Authors:** Muhammad Waqas Khalid, Rajib Ahmed, Ali K. Yetisen, Bader AlQattan, Haider Butt

**Affiliations:** 1 Nanotechnology Laboratory, School of Engineering, University of Birmingham, Birmingham, B15 2TT UK; 2 Harvard-MIT Division of Health Sciences and Technology, Harvard University and Massachusetts Institute of Technology, Cambridge, MA 02139 USA

Correction to: *Scientific Reports* 10.1038/s41598-017-10790-4, published online 06 September 2017

In the original version of this Article, the authors re-used a paragraph in the Results and Discussion section without a proper attribution to the source (ref. 40).

In the Results and Discussion section, under subheading ‘Phase Conjugate Nanostructure Recording’:

“For materials exposed to nanosecond laser pulses, the principal mechanisms of material removal are vaporization and phase explosion. These both phenomena are thermal in nature, as the pulse duration is greater than the phonon-electron relaxation time of the target^37^. When exposed to nanosecond laser pulses of high intensity, the surface of target materials may be heated above the equilibrium boiling temperature toward the critical temperature, T_c_. The emission of particles by normal vaporization is a function of the surface temperature and equilibrium vapour pressure, but is not accompanied by a temperature threshold^38^. With sufficient laser fluence, the target surface temperature may reach 0.8 T_c_, where a dielectric transition takes place and the electrical and thermal conductivities fall by several orders of magnitude^39^. During this process, the surface layer becomes partially transparent and large fluctuations in density occur. With additional absorption of energy from the laser beam, the target surface temperature approaches 0.9 T_c_ and the nucleation rate of vapor bubbles rises dramatically, leading to the ejection of liquid droplets and vapors by explosive boiling, or phase explosion^40^.”

now reads:

“For materials exposed to nanosecond laser pulses, the principal mechanisms of material removal are vaporization and phase explosion^37–39^, which are discussed in detail by Lutey *et al*.^40^”

The authors apologize for the error.

