# Supplementary material for: Holographic Writing of Ink-Based Phase Conjugate Nanostructures via Laser Ablation
Source: Sci Rep. 2017 Sep 6;7:10603. doi: 10.1038/s41598-017-10790-4 (PMC5587581; doi:10.1038/s41598-017-10790-4)
Supplement: Supplementary file 1 — Supporting Information [file 41598_2017_10790_MOESM1_ESM.pdf]

SUPPORTING INFORMATION

Holographic Writing of Ink-Based Phase Conjugate Nanostructures *via* Laser Ablation

Muhammad Waqas Khalid,[1] *Rajib Ahmed,*[1] *Ali K. Yetisen,*[2] *Bader AlQattan,*[1] *and Haider Butt*[1,*]

[1] Nanotechnology Laboratory, School of Engineering, University of Birmingham, Birmingham B15 2TT, UK.

[2] Harvard-MIT Division of Health Sciences and Technology, Harvard University and Massachusetts Institute of Technology, Cambridge, MA, 02139, USA

*E-mail: h.butt@bham.ac.uk, Tel.: +441214158623

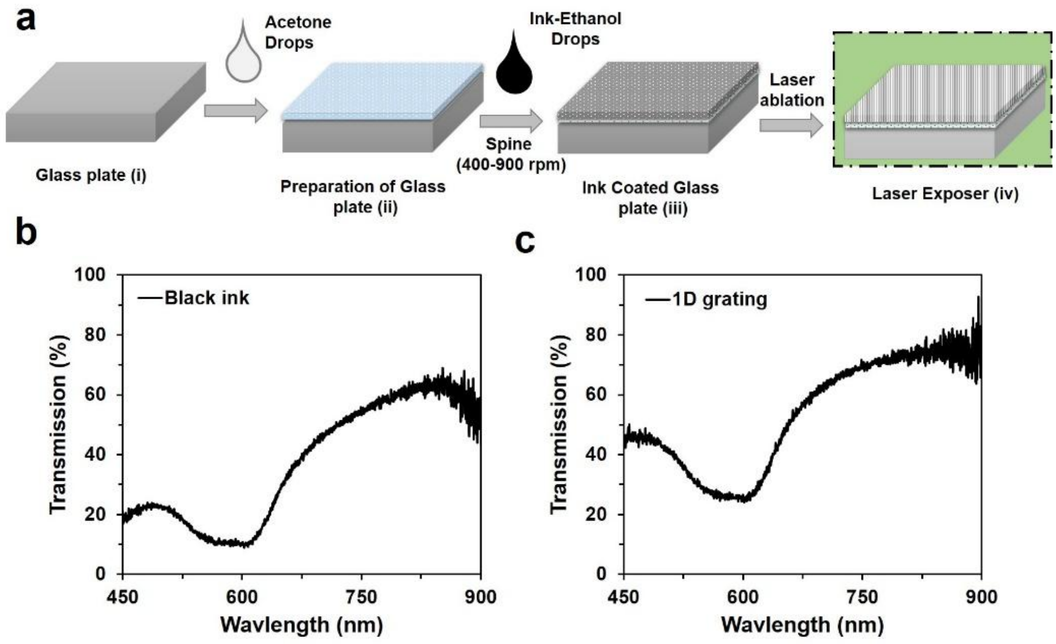

Figure S1: (a) Sample preparation. (b,c) Transmission property of black ink and 1D grating.

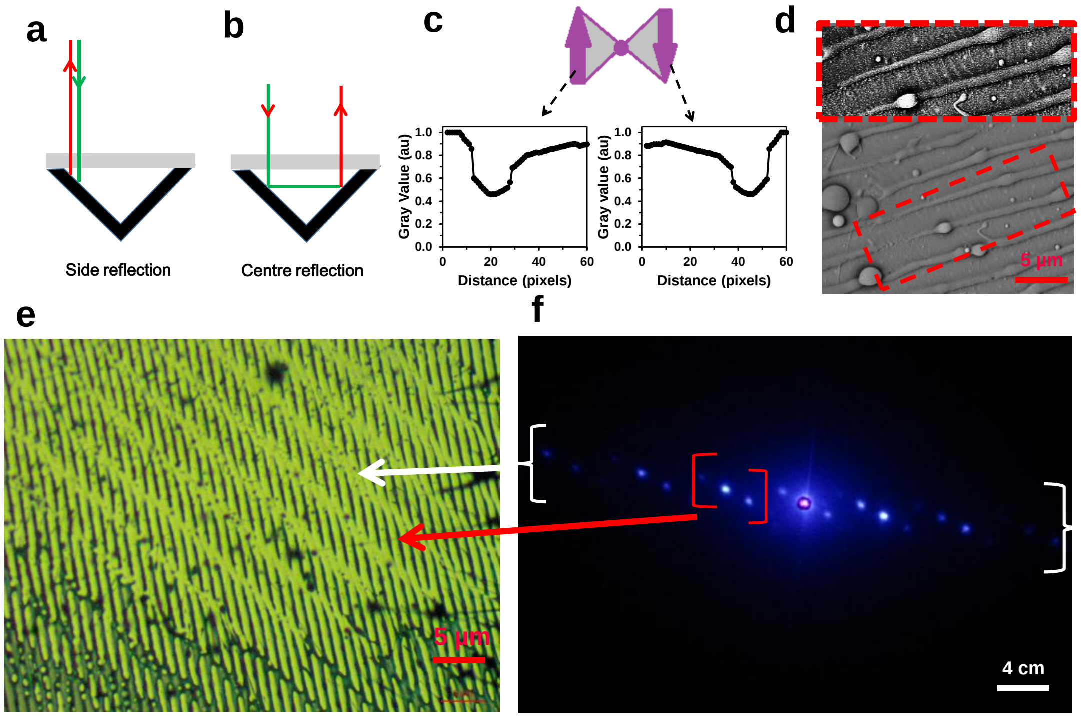

Figure S2: (a,b) Light illumination at side and center of the CCR. (c) Optical phase conjugation. (d) SEM image of the conjugated patterns. (e,f) Light diffraction property of the conjugated structures through violet light illumination.

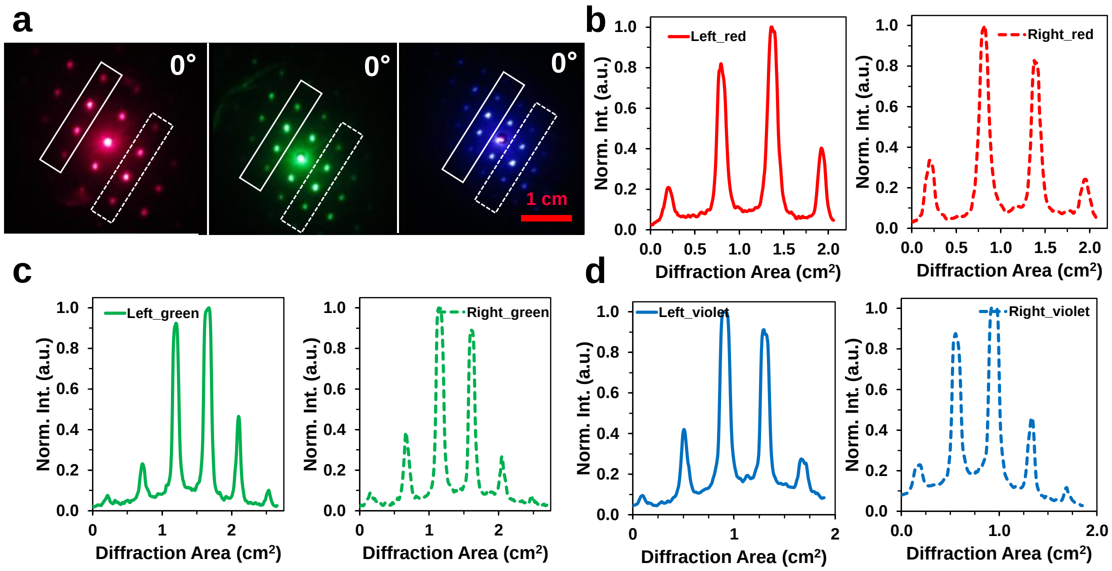

Figure S3: (a) Conjugate diffraction patterns with 0° tilted angle. (b-d) left and right conjugation plots with red, green, and violet light through normal illumination.

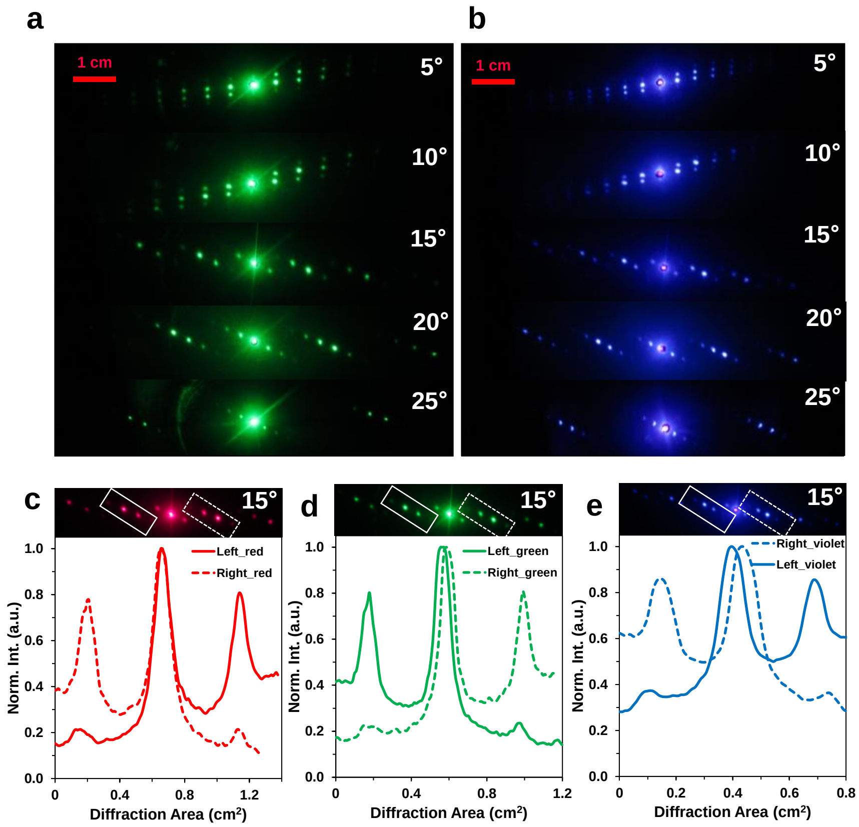

Figure S4: (a,b) Conjugate diffraction patterns with 5-25° tilted angles. (c-e) left and right conjugation plots of 15° tilted sample with red, green, and violet light through normal illumination.

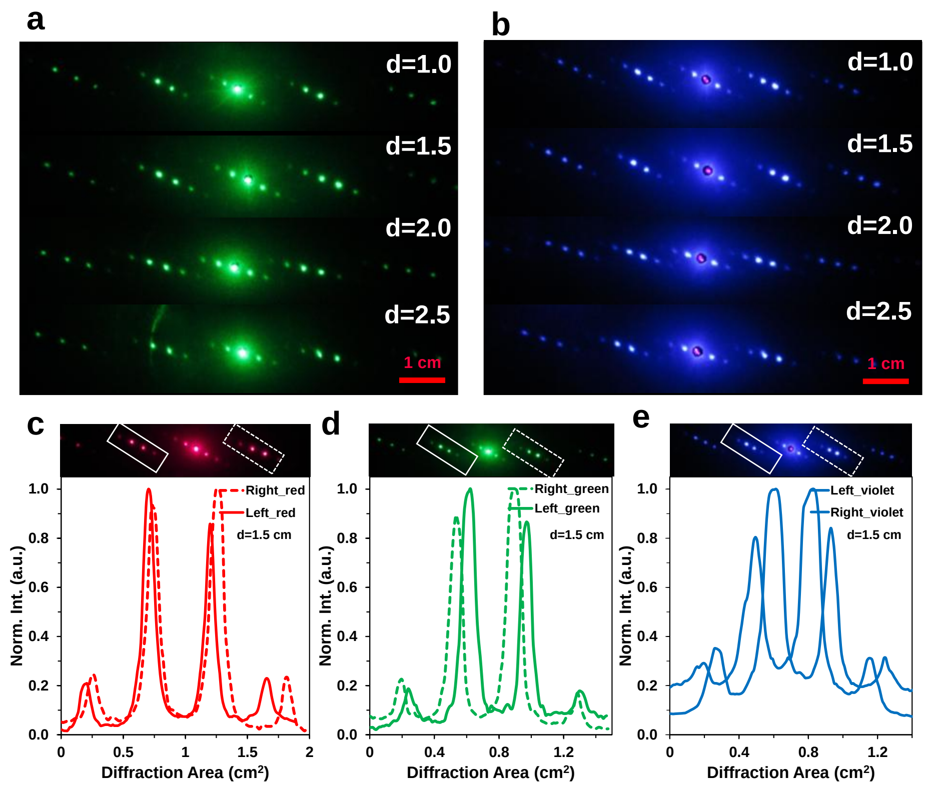

Figure S5: (a,b) Conjugated diffraction patterns through green and violet light illumination with distance variation from 1-2.5 cm. (c-e) left and right conjugation plots of 15° tilted sample with red, green, and violet light through normal illumination.

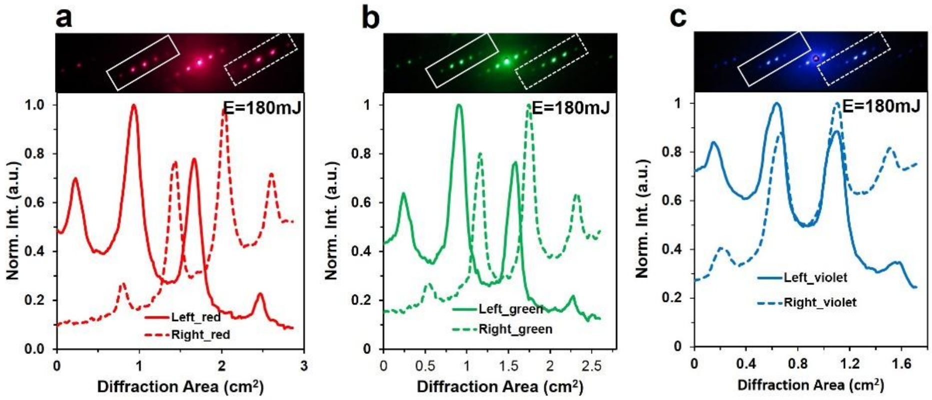

Figure S6: (a-c) Left and right conjugation plots of the recorded sample (Energy, *E*=180mj) through red, green, and violet light normal illumination.

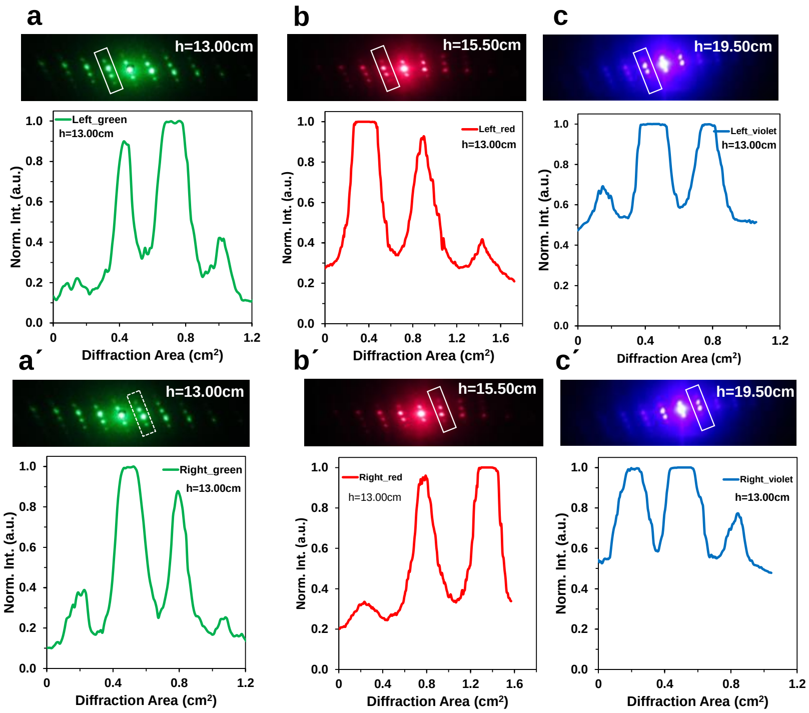

Figure S7: (a-c) Left and right conjugation plots of the recorded sample at working height, h=13.00, 15.50, and 19.50 cm through red, green, and violet light normal illumination.

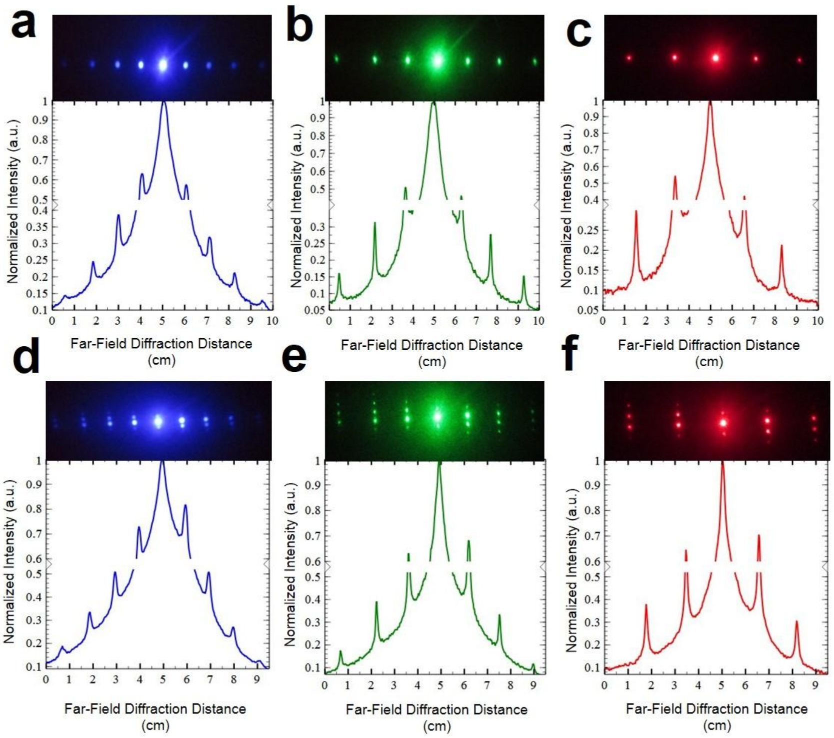

Figure S8: (a-c) conventional, and (d-f) conjugate diffraction plots along horizontal direction through red, green, and violet light normal illumination.
